# A survey on COVID-19 vaccine acceptance and concern among Malaysians

**DOI:** 10.1186/s12889-021-11071-6

**Published:** 2021-06-12

**Authors:** S. A. R. Syed Alwi, E. Rafidah, A. Zurraini, O. Juslina, I. B. Brohi, S. Lukas

**Affiliations:** Department of Family Medicine, Faculty of Medicine and Health Sciences, Universiti Malaysia Sarawak, 94300 Kota Samarahan, Sarawak Malaysia

**Keywords:** COVID-19, Vaccine, Acceptance, Concern

## Abstract

**Background:**

Vaccination is an effective way to curtail the burden of COVID-19 in which success depends on a high acceptance of the vaccine. However, addressing concerns among vaccine-hesitant individuals is essential to avoid failure of the immunisation programme. This study sought to assess the concerns and acceptance rates regarding the COVID-19 vaccine among Malaysians.

**Methods:**

An online questionnaire was distributed to 1411 respondents via a snowball sampling method among Malaysians aged 18 years and above.

**Results:**

The majority of the respondents were young adults (40.7%), female (62.8%), Malay (63.8%), Muslim (72.3%), married (52.9%), with tertiary education (86.8%) and without medical illness (85%). Social media (97.4%) was the primary source of information regarding COVID-19. The overall acceptance rate was high (83.3%), with the lowest rates among the elderly aged 60 years and above (63.4%) and pensioners (64.6%). Hesitance was caused by concerns regarding side effects (95.8%), safety (84.7%), lack of information (80.9%), effectiveness (63.6%) and religious (20.8%) and cultural factors related to the COVID-19 vaccine (6.8%). Respondents with diabetes mellitus (24.7%) and hypercholesterolemia (23%) were more hesitant to accept the COVID-19 vaccine, at 16.1 and 15.8%, respectively. Predictors of COVID-19 vaccine hesitance were age, religion, and current residence.

**Conclusions:**

The results indicate a high rate of acceptance of the COVID-19 vaccine among Malaysians. Thus, the Malaysian government and other related agencies should increase their campaign and prepare to implement the COVID-19 mass immunisation programme among Malaysians. However, despite the high acceptance rate, it remains important to address concerns among hesitant individuals by building trust in vaccine safety and effectiveness through adequate information regarding the vaccine.

**Supplementary Information:**

The online version contains supplementary material available at 10.1186/s12889-021-11071-6.

## Background

The COVID-19 pandemic caused by severe acute respiratory syndrome coronavirus 2 (SARS-CoV-2) has infected more than 108 million people in over 150 countries. In Malaysia, as of February 15, 2021, more than 261,805 confirmed cases with 958 deaths had been reported [1, 2]. The pandemic continues to threaten the healthcare system with catastrophic economic, education, and social consequences worldwide [3, 4].

Currently, no curative treatment exists for COVID-19 infection [5–7]. Therefore, a safe and effective prophylactic vaccine is urgently needed to contain the pandemic, which has had devastating medical, economic, and social repercussions [8]. To date, several vaccines have been developed and approved for emergency immunisation [9–11]. This has given a glimpse of hope for preventing the spread of COVID-19 infection. Countries and governments worldwide have spent billions of dollars in preparing to immunise the population of their countries. In Malaysia, the government has announced an initial purchase of vaccines amounting to RM 3 billion [12].

Vaccination programmes can lead to herd immunity without requiring a substantial proportion of the community to be infected. However, such immunity requires a sufficient proportion of the population to be vaccinated. While vaccination is effectively recognised as an effective way to reduce and eliminate the burden of COVID-19, its effectiveness depends on the population’s willingness to be vaccinated. Immunisation programmes are only successful when there is a high acceptance rate of the vaccine [13–16].

Studies from other countries have identified many factors that influence the acceptance of the COVID-19 vaccine. These include risk perception of the disease, perception of vaccine safety and efficacy, general vaccination attitudes. Past vaccination history, doctors’ recommendation, vaccination costs, vaccination convenience and sociodemographic characteristics [16, 17]. Governments, public health officials and advocacy groups must be prepared to address hesitancy and built vaccine literacy so the public will accept immunisation when appropriate [14].

A few studies have explored the acceptance rates and determinants of the COVID-19 vaccine. One study conducted among healthcare workers (HCW) in China showed high acceptance of the vaccine as compared to the general population. Studies conducted in Saudi Arabia, Indonesia, the European Union, and the United States among the general population showed a high degree of acceptance of the COVID-19 vaccine. However, another study conducted by Decanar Ajumbo et al. (2020) among Western Ugandans showed only average acceptance of the COVID-19 vaccine among the general population [15–19].

This study sought to assess the acceptance of the COVID-19 vaccine among the general population of Malaysia. Understanding the perspectives of the population is critical for the government and relevant agencies so they may formulate the best approach to implement the COVID-19 vaccination programme in Malaysia.

## Methods

This cross-sectional study was conducted from December 23–29, 2020, after approval from the Research and Ethics Committee, Faculty of Medicine and Health Sciences, Universiti Malaysia Sarawak (approval reference: NO RUJUKAN ETIKA FME/21/40). All research methods were performed in accordance with the relevant guidelines and regulations of the Declaration of Helsinki. The target participants were Malaysian adults aged 18 years and above who could read and understand Bahasa Malaysia or English. Due to the limitations in employing face-to-face methods during an active outbreak, the data were collected using the Google Forms platform via an online questionnaire. A snowball sampling strategy was used to distribute the online questionnaire via social media (WhatsApp, Facebook, Twitter). First, 50 primary recipients were recruited. These participants were then asked to share the questionnaire link to individuals in their social circles (aged 18 years and above). These social media platforms were chosen because they are widely used among the Malaysian population across sociodemographic characteristics.

The minimum sample size was calculated using OpenEpi Version 3. In 2019, the Malaysian population of adults aged 18 years and above was around 21.8 million [20]. The minimum sample size calculated with a 95% confidence interval was 384. A total of 1411 completed responses were collected.

### Questionnaire on COVID-19 vaccine: acceptance and concerns

The questionnaire was developed via expert group discussion and literature reviews [15–18]. The survey form consisted of 14 questions covering sociodemographic characteristics (Questions 1 to 10), medical illnesses (Question 11), source of information regarding COVID-19 (Question 12), acceptance of COVID-19 vaccine (Question 13) and concerns regarding the COVID-19 vaccine (Question 14). All respondents were required to answer Questions 1–13. Question 14 was only answered by respondents who responded ‘no’ to Question 13.

Sociodemographic characteristics (Questions 1–10) included current residency, place of birth, age in years, gender, race, religion, marital status, education level, occupation and monthly income. Medical illnesses (Question 11) included diabetes, hypertension, hypercholesterolemia, respiratory disease, renal disease, heart disease and cancer. Source of information regarding COVID-19 (Question 12) included social media, mass media, HCWs and friends/family/neighbour. Question 14 addressed the concerns regarding the COVID-19 vaccine, such as a lack of information regarding the vaccine, side effects, safety, effectiveness, COVID-19 is not dangerous, fear of injection, against vaccination in general, religious reasons, cultural reasons and belief in traditional remedies. For Questions 11, 12, and 14, respondents could choose more than one answer but only one answer for Question 13. The answer responses for Questions 11, 12, 13 and 14 were in dichotomous ‘yes’ or ‘no’ format.

The original questionnaire was developed in English and later translated to Bahasa Malaysia using forward and back translation. A pilot study was conducted among the general population through snowball sampling of 76 individuals, 20% of the total calculated sample size of 384, to ensure the questions were clearly written, easily understood and unambiguous. A reliability test was conducted for Question 14 to determine the internal consistency of 10 items listed measuring COVID-19 vaccine concerns, resulting in a Cronbach’s alpha of 0.6.

### Data analysis

Data analysis was done using the Statistical Package for Social Science version 22.0. Descriptive analyses were used for sociodemographic and categorical data. The variables associated with COVID-19 vaccine acceptance and hesitancy were analysed using bivariate analysis. Further analyses were conducted to identify predictors of COVID-19 vaccine hesitancy using multiple logistic regression. A *p*-value of less than .05 and a confidence interval of 95% was considered statistically significant.

Another analysis to determine significant concerns among hesitant respondents was performed using the Relative Importance Index (RII), as shown in Table 3. Ten items were listed to measure concerns among hesitant respondents with dichotomous responses of ‘yes’ or ‘no’. A score of 2 was given to ‘yes’ responses and 1 to ‘no’ responses to calculate the RII for each item using the formula below:

$$ \mathrm{RII}=\Sigma\mathrm{W}/\left(\mathrm{A}\ast \mathrm{N}\right) $$

$$ \mathrm{W}=\mathrm{weighting}\ \mathrm{for}\ \mathrm{each}\ \mathrm{item} $$

$$ \mathrm{A}=2\left(\mathrm{the}\ \mathrm{highest}\ \mathrm{weight}\right) $$

$$ n=236\ \left(\mathrm{total}\ \mathrm{number}\ \mathrm{of}\ \mathrm{hesitant}\ \mathrm{respondents}\right) $$

## Results

A total of 1411 respondents participated in this study. Table 1 provides an overview of their sociodemographic characteristics. Almost half of the respondents (40.7%) were young adults (18–29 years old), whereas the elderly aged 60 years and above were the minority (5.8%). The majority of respondents were female (62.8%), Malay (63.8%), Muslim (72.3%), married (52.9%) and had tertiary education (86.8%). Respondents came from various occupation sectors, including students (25.4%), education (16.6%), medical and health (15.6%) and management and administrative (10%). Other occupations such as services (8.4%), industrial and manufacturing (4.5%), security and defence (2.2%), construction (2.2%), other occupation (2%), pensioner (5.8%) and unemployed (7%) contributed to the minority group. About one third of the respondents were in the lowest monthly income category of RM 0–1200 (35.5%), followed by RM 1201–4000 (25.2%) and RM 4000–8000 (25.1%). Almost half of the respondents resided in Sarawak state (48.7%).

**Table 1:** Sociodemographic characteristics among respondents (*N* = 1411)

Sociodemographic characteristics	***n***	%
Age (year)		
18–29	574	40.7
30–39	341	24.2
40–49	246	17.4
50–59	168	11.9
60 and above	82	5.8
Gender		
Male	525	37.2
Female	886	62.8
Race		
Malay	901	63.8
Chinese	159	11.3
Indian	43	3
Iban	78	5.5
Bidayuh	56	4
Melanau	46	3.3
Orang Ulu	29	2.1
Kadazan	23	1.6
Dusun	23	1.6
Others	53	3.8
Religion		
Islam	1020	72.3
Christianity	271	19.2
Buddhism	80	5.7
Other	40	2.8
Marital status		
Single	614	43.5
Married	746	52.9
Divorced	31	2.2
Widow/Widower	20	1.4
Education		
Less than tertiary education	185	13.1
Tertiary education	1226	86.9
Occupation		
Medical & Health	220	15.6
Education	234	16.6
Security & Defence	31	2.2
Industrial & Manufacturing	63	4.5
Construction	31	2.2
Management & Administrative	141	10
Services	119	8.4
Student	358	25.4
Pensioner	82	5.8
Unemployed	99	7
Others	33	2.4
Monthly income (RM)		
0–1200	501	35.5
1201–4000	356	25.2
4001–8000	354	25.2
> 8000	200	14.2
Current residence		
Sarawak	687	48.7
Sabah & WP Labuan	102	7.23
Northern Zone*	118	8.36
Middle Zone*	336	23.8
Southern Zone*	117	8.29
East Coast Zone*	51	3.62

*Northern Zone: Kedah, Perak, Pulau Pinang, Perlis

*Middle Zone: Selangor, WP Kuala Lumpur, WP Putrajaya

*Southern Zone: Negeri Sembilan, Melaka, Johor

*East Coast Zone: Kelantan, Terengganu, Pahang

More than 85% of the respondents reported having no known medical illness (Table 2). A small percentage of respondents had hypercholesterolaemia (13.3%), hypertension (12.8%), diabetes mellitus (6.9%), respiratory disorder (4%), heart disease (2%), renal disorder (1.3%) and cancer (0.6%).

**Table 2:** Medical illnesses, source of information, acceptance and concerns regarding COVID-19 vaccine among respondents (*N* = 1411)

Medical illnesses		***n***	%
Diabetes mellitus	Yes	97	6.9
	No	1314	93.1
Hypertension	Yes	180	12.8
	No	1231	87.2
Hypercholesterolemia	Yes	187	13.3
	No	1224	86.7
Respiratory disorder	Yes	56	4
	No	1355	96
Renal disorder	Yes	19	1.3
	No	1392	98.7
Heart disease	Yes	28	2
	No	1383	98
Cancer	Yes	8	0.6
	No	1403	99.4
**Source of information regarding COVID-19**			
Social media	Yes	1375	97.4
	No	36	2.6
Mass media	Yes	1327	94
	No	84	6
Healthcare worker	Yes	844	59.8
	No	567	40.4
Others (family, friend, neighbour etc.)	Yes	1119	79.3
	No	292	20.7
**Acceptance of the COVID-19 vaccine**			
Acceptance	Yes	1175	83.3
	No	236	16.7
**Concerns regarding the COVID-19 vaccine**			
Lack of information	Yes	191	80.9
	No	45	19.1
Side effects	Yes	226	95.8
	No	10	4.2
Not safe	Yes	200	84.7
	No	36	15.3
Not effective	Yes	150	63.6
	No	86	36.4
COVID-19 is not dangerous	Yes	53	22.5
	No	183	77.5
Fear of injection	Yes	38	16.1
	No	198	83.9
Against vaccines in general	Yes	66	28
	No	170	72
Religious reasons	Yes	49	20.8
	No	187	79.2
Cultural reasons	Yes	16	6.8
	No	220	93.2
Believe in traditional remedies	Yes	42	17.8
	No	194	82.2

### Information regarding COVID-19 and COVID-19 vaccine hesitancy

Most respondents acquired information regarding COVID-19 through social media (97.4%), mass media (94%), friends and family (79.3%) and HCWs (59.8%) (Table 2). COVID-19 vaccine acceptance in Malaysia (83.3%) was substantially higher than hesitance (16.7%). Hesitant respondents reported they were concerned about the side effects (95.8%), safety (84.7%) and lack of information regarding the vaccine (80.9%). Another concern by more than half of the respondents was the vaccine’s effectiveness (63.6%). Slightly more than one quarter were against vaccination in general (28%), whereas slightly less than one quarter believed COVID-19 is not dangerous (22.5%). Minority groups were not willing to accept vaccination due to religious (20.8%) and cultural (6.8%) reasons, belief in traditional remedies (17.8%) and fear of injection (16.1%).

The RII for concerns regarding the COVID-19 vaccine among hesitant respondents indicated the most important concerns were side effects (RII = 0.98), followed by safety (RII = 0.92), lack of information (RII = 0.90) and effectiveness (RII = 0.82) of the vaccine (Table 3).

**Table 3:** Relative Important Index (RII) for concerns regarding COVID-19 vaccine among hesitant respondents (*n* = 236)

Item	Concerns regarding COVID-19 vaccine	RII
1	Lack of information	0.90
2	Side effects	0.98
3	Not safe	0.92
4	Not effective	0.82
5	COVID-19 is not dangerous	0.61
6	Fear of injection	0.58
7	Against vaccines in general	0.64
8	Religious reasons	0.60
9	Cultural reasons	0.53
10	Believe in traditional remedies	0.59

### Factors associated with COVID-19 vaccine hesitancy

As shown in Table 4, sociodemographic factors significantly associated with COVID-19 vaccine hesitance were age (*p* < .001), race (*p* = .025), marital status (*p* = .001), occupation (*p* < .001), monthly income (*p* = .044), current residence (*p* = .018) and having diabetes (*p* = .028) and hypercholesterolemia (*p* = .014). COVID-19 vaccine hesitance was ignificantly different across age groups, with the highest hesitance seen among the oldest age group of 60 years and above (36.6%). Among all races in Malaysia, a substantial proportion of COVID-19 vaccine hesitance was seen among Kadazan (30.4%), Indian (27.9%), Dusun (26.1%), Orang Ulu (24.1%) and other indigenous and non-Malaysian groups (22.6%). Furthermore, respondents who were married or divorced were more hesitant to get the COVID-19 vaccine than single respondents (12.1%) and widows/widowers (15%). Pensioners (35.4%) were the most hesitant to accept the COVID-19 vaccine across all occupations. Hesitance was highest among respondents with monthly incomes of RM 4000 and above.

**Table 4:** Factors associated with COVID-19 vaccine hesitance among respondents (*N* = 1411)

Variable	*n*	Acceptance *n* (%)	Hesitance *n* (%)	*P* value^a^
**Age (year)**				**<.001**
18–29	574	507 (88.3)	67 (11.7)	
30–39	341	283 (83)	58 (17)	
40–49	246	199 (80.9)	47 (19.1)	
50–59	168	134 (79.8)	34 (20.2)	
60 and above	82	52 (63.4)	30 (36.6)	
**Gender**				.747
Male	525	435 (82.9)	90 (17.1)	
Female	886	740 (83.5)	146 (16.5)	
**Race**				**.025**
Malay	901	763 (84.7)	138 (15.3)	
Chinese	159	126 (79.2)	33 (20.8)	
Indian	43	31 (72.1)	12 (27.9)	
Iban	78	72 (92.3)	6 (7.7)	
Bidayuh	56	47 (83.9)	9 (16.1)	
Melanau	46	40 (87)	6 (13)	
Orang Ulu	29	22 (75.9)	7 (24.1)	
Kadazan	23	16 (69.6)	7 (30.4)	
Dusun	23	17 (73.9)	6 (26.1)	
Other Indigenous & Non-Malaysian	53	41 (77.4)	12 (22.6)	
**Religion**				.081
Islam	1020	857 (84)	163 (16)	
Christianity	271	228 (84.1)	43 (15.9)	
Buddhism	80	61 (76.3)	19 (23.8)	
Other	40	29 (72.5)	11 (27.5)	
**Marital Status**				**.001**
Single	614	540 (87.9)	74 (12.1)	
Married	746	594 (79.6)	152 (20.4)	
Divorced	31	24 (77.4)	7 (22.6)	
Widow/Widower	20	17 (85)	3 (15)	
**Education**				.842
Less than tertiary	185	155 (83.8)	30 (16.2)	
Tertiary education	1226	1020 (83.2)	206 (16.8)	
**Occupation**				**<.001**
Medical & Health	220	177 (80.5)	43 (19.5)	
Education	234	188 (80.3)	46 (19.7)	
Security & Defence	31	27 (87.1)	4 (12.9)	
Industrial &	63	55 (87.3)	8 (12.7)	
Manufacturing				
Construction	31	25 (80.6)	6 (19.4)	
Management & Administrative	141	123 (87.2)	18 (12.8)	
Services	119	96 (80.7)	23 (19.3)	
Student	358	324 (90.5)	34 (9.5)	
Pensioner	82	53 (64.6)	29 (35.4)	
Unemployed	99	82 (82.8)	17 (17.2)	
Other	33	25 (75.8)	8 (24.2)	
**Monthly Income (RM)**				**.044**
0–1200	501	434 (86.6)	67 (13.4)	
1201–4000	356	297 (83.4)	59 (16.6)	
4001–8000	354	282 (79.7)	72 (20.3)	
> 8000	200	162 (81)	38 (19)	
**Current Residence**				**.018**
Sarawak	687	578 (84.1)	109 (15.8)	
Sabah & WP Labuan	102	75 (73.5)	27 (26.5)	
Northern Zone*	118	97 (82.2)	21 (17.8)	
Middle Zone*	336	289 (86)	47 (14)	
Southern Zone*	117	99 (84.6)	18 (15.4)	
East Coast Zone*	51	37 (72.5)	14 (27.5)	
**Diabetes Mellitus**				**.028**
No	1314	1102 (83.9)	212 (16.1)	
Yes	97	73 (75.3)	24 (24.7)	
Hypertension				.140
No	1231	1032 (83.8)	199 (16.2)	
Yes	180	143 (79.4)	37 (20.6)	
**Hypercholesterolemia**				**.014**
No	1224	1031 (84.2)	193 (15.8)	
Yes	187	144 (77)	43 (23)	
Respiratory Disorder				.184
No	1355	1132 (83.5)	223 (16.5)	
Yes	56	43 (76.8)	13 (23.2)	
Renal Disorder				.543^b^
No	1392	1160 (83.3)	232 (16.7)	
Yes	19	15 (78.9)	4 (21.1)	
Heart Disease				.301^b^
No	1383	1154 (83.4)	229 (16.6)	
Yes	28	21 (75)	7 (25)	
Cancer				1^b^
No	1403	1168 (83.3)	235 (16.7)	
Yes	8	7 (87.5)	1 (12.5)	
Social Media				.072
No	36	26 (72.2)	10 (27.8)	
Yes	1375	1149 (83.6)	226 (16.4)	
Mass Media				.774
No	84	69 (82.1)	15 (17.9)	
Yes	1327	1106 (83.3)	221 (16.7)	
Healthcare Workers				.981
No	567	472 (83.2)	95 (16.8)	
Yes	844	703 (83.3)	141 (16.7)	
Friends, family, etc.				.746
No Yes	292	245 (83.9)	47 (16.1)	
	1119	930 (83.1)	189 (16.9)	

^a^Chi-square test for independence ^b^Fisher’s exact test

*Northern Zone: Kedah, Perak, Pulau Pinang, Perlis

*Middle Zone: Selangor, WP Kuala Lumpur, WP Putrajaya

*Southern Zone: Negeri Sembilan, Melaka, Johor

*East Coast Zone: Kelantan, Terengganu, Pahang

The proportion of hesitance between respondents in different states in Malaysia was also significantly different (*p* = .018). Respondents residing in the East Coast Zone of Peninsular Malaysia and Sabah & WP Labuan had the highest proportion of hesitance (27.5 and 26.5%, respectively). Respondents with diabetes mellitus (24.7%) and hypercholesterolemia (23%) were more hesitant to accept the COVID-19 vaccine than those without (16.1 and 15.8%, respectively).

### Predictors of COVID-19 vaccine hesitance

Further analysis using multiple logistic regression was performed to determine predictors of COVID-19 vaccine hesitance among respondents (Table 5). The model containing all 20 independent variables was statistically significant, 풳^2^ = 59.19 *p* < .001. The model as a whole explained between 4% (Cox and Snell R Square) and 7% (Nagelkerke R Square) of variance in COVID-19 vaccine hesitance and correctly classified 83.2% of the cases.

**Table 5:** Predictors of COVID-19 vaccine hesitance among respondents (*N* = 1411)

	Crude OR	Adj. *OR*	(95% CI *OR*)	*P* value^a^
Age (years)				
18–29				
30–39	1.55	1.60	1.09, 2.36	.017
40–49	1.79	1.99	1.31, 3.02	.001
50–59	1.92	2.04	1.28, 3.23	.003
60 and above	4.37	5.22	3.06, 8.90	<.001
Religion				
Islam	1.00	1.00		
Christianity	0.99	1.08	0.73, 1.60	.700
Buddhism	1.64	2.01	1.15, 3.53	.015
Other	1.99	2.67	1.26, 5.64	.010
Current Residence				
Sarawak	1.00	1.00		
Sabah & WP Labuan	1.91	2.22	1.35, 3.66	.002
Northern Zone*	1.15	1.07	0.62, 1.83	.812
Middle Zone*	0.86	0.82	0.55, 1.21	.308
Southern Zone*	0.96	1.01	0.57, 1.77	.985
East Coast Zone*	2.01	2.55	1.30, 5.01	.007

*Adj. OR* Adjusted odds ratio

*Northern Zone: Kedah, Perak, Pulau Pinang, Perlis

*Middle Zone: Selangor, WP Kuala Lumpur, WP Putrajaya

*Southern Zone: Negeri Sembilan, Melaka, Johor

*East Coast Zone: Kelantan, Terengganu, Pahang

As shown in Table 5, only three variables made a statistically significant contribution to the model. The strongest predictor of COVID-19 vaccine hesitance was age. Respondents aged 60 years and above were five times more hesitant and respondents aged 30 to 59 years were two times more hesitant to take the COVID-19 vaccine when compared with the younger age group of 18 to 29 years after controlling for all other factors in the model. Other predictors of COVID-19 hesitance were religion and current residence. Individuals who reported following Buddhism and other religions were twice as hesitant to take the COVID-19 vaccine compared to those following Islam. Meanwhile, respondents from Sabah, WP Labuan, and the East Coast Zone in Peninsular Malaysia were twice more hesitant to take the COVID-19 vaccine than individuals residing in Sarawak.

## Discussion

Vaccination is recognised as an effective way to reduce and eliminate the burden of COVID-19. However, the success of a vaccination programme depends on the willingness of the population to be vaccinated. This study used an online self-administered questionnaire and collected responses across Malaysia. Out of 1411 respondents, the acceptance rate of the COVID-19 vaccine in Malaysia was 83.3%, much higher than the hesitance rate (16.7%). This acceptance rate corresponds to studies conducted among the general population in Indonesia, China, Europe and Saudi Arabia [15–18].

For this study, age, race, marital status, current residence, monthly income, occupation and medical condition (diabetes mellitus and hypercholesterolaemia) significantly influenced vaccine acceptance (*p* < .05) (Table 4). In contrast, studies in China and Saudi Arabia only showed gender and marital status as significantly influencing vaccine acceptance [16, 18]. Studies from Indonesia did not show any significant association between sociodemographic characteristics and vaccine acceptance, except being an HCW [15]. The difference between these findings might be due to different methodology and sociodemographic characteristics among the participants in the study.

Further analysis using multiple logistic regression identified significant predictors for COVID-19 vaccine hesitancy, including age, religion and current residence. Respondents aged 60 years and above were five times more hesitant to take the vaccine, followed by respondents aged 30 to 59 who were twice as likely to hesitate when compared to the youngest group of respondents aged 18 to 29 years. This finding contrasts to those of Robertson et al., who found that UK residents aged 25 to 34 years were more hesitant to take the vaccine compared to those aged 45 to 54 years [20]. Our findings also indicate that Buddhists are twice as likely to hesitate than Muslims. Recent studies among UK and US citizens have not shown the significance of religion as a predictor of vaccine hesitancy [20, 21]. Additionally, respondents who resided in the East Coast Zone of Peninsular Malaysia and Sabah & WP Labuan were twice as likely to hesitate than respondents who resided in Sarawak. Notably, a recent local study did not investigate residency across different states in Malaysia as a predictor of vaccine hesitancy [22]. Any interpretation of these findings should consider the sociodemographic characteristics of respondents, as the sample was predominantly comprised of Sarawakians, Malays and Muslims.

It was expected that the majority of the respondents would be from younger age groups, and, indeed, only 5.8% were from the elderly population. This could be due to the sampling method and how the questionnaire was circulated, as the elder age group might have limited access to smartphones and the internet. In the occupation group, students were more accepting compared with those working in medical and health occupations. This finding was different from the Indonesia study that showed HCWs were twice likely to accept the COVID-19 vaccine [15]. Although this study did not evaluate factors influencing COVID-19 vaccine acceptance, it is hypothesised that the reasons for potential acceptance are similar to those in other parts of the world, namely when the vaccine is generally available, if it is recommended by the employer and confidence in the development and safety of the vaccine [14, 23].

In a study conducted by researchers from the Institute of Clinical Research in July 2020 (before the availability of the COVID-19 vaccine), the vaccine’s acceptance rate was much higher at 93.2%, whereas the refusal rate was only 6.8% [24]. Factors that predicted COVID-19 vaccine acceptance were perception of the COVID-19 situation, COVID-19 as a threat to health and the susceptibility to infection. Notably, the acceptance rate was lower in the current study despite the knowledge that the vaccine is already developed and waiting to be available in Malaysia. A recent non-peer-reviewed survey done among 212,000 Malaysians had a much lower acceptance rate at only 67% [25].

Herd immunity is also known as ‘population immunity’. For herd immunity to occur, the population coverage required through vaccination varies across diseases and is dependent on the basic reproduction number (R_0_), vaccine efficacy and duration of immunity [26, 27]. The proportion of the population that must be vaccinated against COVID-19 to begin inducing herd immunity is unknown [28]. Thus, the challenge to determine the sufficient proportion of the population to create such immunity by mass vaccination remains. Nevertheless, the larger the number of vaccinated individuals, the better the immune coverage. Experts in Malaysia estimated that the proportion of the population that would need to be vaccinated might be as high as 80 to 90%, as the R_0_ number for some of the clusters in the current third wave seems to be relatively high [29].

Vaccine hesitancy was named as one of the top 10 threats to global health by the WHO in 2019. This was in response to a reduction in global immunisation rates for the measles, mumps and rubella vaccine, which slipped to 85% compared to the required target of 95% and led to numerous measles outbreaks [30]. The reluctance and refusal of the COVID-19 vaccine is a global problem. Furthermore, to have a substantial population immunised to achieve herd immunity, reasons for vaccine hesitancy must be addressed. In this study, the rate for COVID-19 vaccine hesitance was 16.7% among 1411 respondents.

The leading cause for hesitancy was fear of the side effects of the vaccine (95.8%), concerns about the safety (84.7%), lack of information (80.9%) and questions about the effectiveness of a new vaccine (63.6%). Further analysis using the RII supported that side effects, safety, lack of information and effectiveness were the crucial concerns among respondents. This finding is similar to studies conducted in other countries that identified concerns over vaccine safety, potential side effects and effectiveness [15, 17].

Public confidence in vaccination to prevent infection may be affected by perceived risks associated with vaccination. This was reflected in the hesitant group, in which 95.8% reported a fear of vaccine side effects as a reason for their refusal. With the COVID-19 vaccine and ongoing trials, the most common side effects reported were pain at the injection site, tiredness, headache, muscle pain, chills, joint pain, and fever, which typically would last for several days [31]. These side effects were noted more common after the second dose on vaccine trials. Although severe side effects, such as anaphylaxis, may occur, these are rare [32]. However, the risks of contracting COVID-19 infection, which may lead to severe complications, outweigh the risks of getting the vaccine.

Other reasons for the COVID-19 vaccine hesitance were being against vaccines in general (28%), the perception that COVID-19 is not dangerous (22.5%), religious reasons (20.8%), belief in traditional remedies (17.8%), fear of injection (16.1%) and cultural reasons (6.8%). Similar findings are seen in the studies conducted in Europe [17]. The reason for vaccine hesitancy is usually due to a lack of trust in the health care professionals and the government to choose an alternative or complementary medicine for their treatment, which cannot replace the vaccine function [33]. Although the number of those against the vaccine, in general, has been relatively small, their opposing view on vaccines must be acknowledged because they may influence others, especially those undecided but aware of the risks of the vaccine [34].

When discussing vaccine hesitancy or refusal, one cannot ignore conspiracy theories. Conspiracy theories increase in times of crisis when people feel threatened, uncertain and insecure – much like the situation created by the COVID-19 pandemic [35]. Anti-vaccine conspiracy theories appear to reduce vaccination intentions by inducing undue concerns about the dangers of vaccines and increasing feelings of powerlessness, disillusionment and mistrust in authorities [36]. Thus, effective measures to reduce the negative impact of conspiracy theories must be implemented. These include having an ethical and responsible mass media with media regulatory authorities on statement guidelines regarding the COVID-19 pandemic, addressing religious elements surrounding the conspiracy theories, stern measures from the healthcare authorities and increasing awareness on COVID-19 to reduce negative perceptions among the general community [37].

In this study, the respondents’ source of information about COVID-19 was mainly from social media (97.4%) and mass media (94.0%). Many reliable websites are providing the public with updates regarding this disease, including information about the vaccines, namely the WHO, Centers for Disease Control and Prevention (CDC) and the Ministry of Health (MOH) [38–40]. Information about this pandemic is also regularly aired across radio, television networks and newspapers, and thus it is quite worrying that 80.9% of the respondents stated that they lacked information about the COVID-19 vaccine. With the robust information available, it can be too overwhelming for the general population. Thus, further intervention needs to be done to convey the relevant health education to enable good knowledge transfer resulting in increased awareness to make informed choices.

Although the acceptance of the COVID-19 vaccine in this study was relatively high, the actual willingness to vaccinate will only be known once the vaccine arrives in Malaysia. As for now, the measures to prevent infection via wearing face masks, physical distancing, proper handwashing and avoidance of crowded and small confined spaces must be emphasised at all times.

### Limitations of the study

Data collection occurred online, which means we may not have reached vulnerable groups, including those with lower socioeconomic background and those who are illiterate. Also, a larger percentage of the respondents were from a single geographic area, which may impact the generalisation of the survey results.

## Conclusions

The acceptance rate of the COVID-19 vaccine among the Malaysian population who participated in this study was high. This corresponds to other studies conducted with Asian and Western populations. The Malaysian government and other related agencies must target their campaign to address the concerns reported here about the vaccine and prepare to implement the mass immunisation programme for COVID-19 for Malaysian citizens. In implementing the nationwide COVID-19 vaccination programme, researchers must continue to investigate vaccine hesitancy and health education must be prepared to address any concerns that may arise during the programme.

## Supplementary Information

**Additional file 1 Appendix 1.** Questionnaire (English version). **Appendix 2.** Questionnaire (Bahasa Melayu version).

## Data Availability

The datasets used and analysed during the current study are available from the corresponding author on reasonable request.

## Abbreviations

%: Percentage
CDC: Centers of Disease Control and Prevention
CI: Confidence interval
COVID-19: Corona virus disease 2019
MOH: Ministry of Health
OR: Odds ratio
*P*-Value: Probability value
WHO: World Health Organization
