# Supplementary material for: A survey on COVID-19 vaccine acceptance and concern among Malaysians
Source: BMC Public Health. 2021 Jun 12;21:1129. doi: 10.1186/s12889-021-11071-6 (PMC8196915; doi:10.1186/s12889-021-11071-6)
Supplement: Supplementary file 1 — Additional file 1 Appendix 1. Questionnaire (English version). Appendix 2. Questionnaire (Bahasa Melayu version). [file 12889_2021_11071_MOESM1_ESM.docx]

Appendix 1: Questionnaire (English version)

COVID-19 VACCINE: ACCEPTANCE & CONCERN

1. Current Residence (Please mark one answer)

|  | Sarawak |
| --- | --- |
|  | Sabah |
|  | Perlis |
|  | Kedah |
|  | Pulau Pinang |
|  | Perak |
|  | Selangor |
|  | Melaka |
|  | Negeri Sembilan |
|  | Johor |
|  | Pahang |
|  | Kelantan |
|  | Trengganu |
|  | Wilayah Persekutuan Putrajaya |
|  | Wilayah Persekutuan Kuala Lumpur |
|  | Wilayah Persekutuan Labuan |
|  | Other; |

1. Place of birth (Please mark one answer)

|  | Sarawak |
| --- | --- |
|  | Sabah |
|  | Perlis |
|  | Kedah |
|  | Pulau Pinang |
|  | Perak |
|  | Selangor |
|  | Melaka |
|  | Negeri Sembilan |
|  | Johor |
|  | Pahang |
|  | Kelantan |
|  | Trengganu |
|  | Wilayah Persekutuan Putrajaya |
|  | Wilayah Persekutuan Kuala Lumpur |
|  | Wilayah Persekutuan Labuan |
|  | Other: |

1. Age (Year) (Please mark one answer)

|  | 18-29 |
| --- | --- |
|  | 30-39 |
|  | 40-49 |
|  | 50-59 |
|  | 60 and above |

1. Gender (Please mark one answer)

|  | Male |
| --- | --- |
|  | Female |

1. Race (Please mark one answer)

|  | Malay |
| --- | --- |
|  | Chinese |
|  | Indian |
|  | Iban |
|  | Bidayuh |
|  | Melanau |
|  | Orang Ulu |
|  | Kadazan |
|  | Dusun |
|  | Murut |
|  | Bajau |
|  | Orang Asli |
|  | Other: |

1. Religion (Please mark one answer)

|  | Islam |
| --- | --- |
|  | Christian |
|  | Hindu |
|  | Buddhist |
|  | Other: |

1. Marital status (Please mark one answer)

|  | Married |
| --- | --- |
|  | Divorced |
|  | Widow or widower |
|  | Single |

1. Education level (Please mark one answer)

|  | Primary |
| --- | --- |
|  | Secondary |
|  | Tertiary (College/University) |
|  | No formal education |

1. Occupation (Please mark one answer)

|  | Medical & Health |
| --- | --- |
|  | Education |
|  | Security & Defense |
|  | Industrial & Manufacturing |
|  | Tourism & Hospitality |
|  | Construction |
|  | Management & Administrative |
|  | Services (e-hailing, food & beverages, etc) |
|  | Student |
|  | Pensioner |
|  | Unemployed |
|  | Other: |

1. Monthly income (RM) (Please mark one answer)

|  | 0-1200 |
| --- | --- |
|  | 1201-4000 |
|  | 4001-8000 |
|  | >8000 |

1. Medical illness (Please answer every statement)

|  | Yes | No |
| --- | --- | --- |
| Diabetes |  |  |
| Hypertension |  |  |
| Hypercholesterolemia |  |  |
| Respiratory disease |  |  |
| Renal disease |  |  |
| Heart disease |  |  |
| Cancer |  |  |

1. Source of information regarding Covid-19 (Please answer every statement)

|  | Yes | No |
| --- | --- | --- |
| Social media (WhatsApp, Telegram, Twitter, Instagram, Facebook, Website etc) |  |  |
| Mass media (TV, Radio, Newspaper) |  |  |
| Healthcare worker |  |  |
| Friends, family, neighbor etc |  |  |

1. If the Malaysian Government provides COVID-19 vaccine for free, do you agree to receive the vaccination? (Please mark one answer)

|  | Yes |
| --- | --- |
|  | No, to proceed to question 14 |

1. What are your concern regarding Covid-19 vaccine? (Please answer every statement)

|  | Yes | No |
| --- | --- | --- |
| Lack of information regarding Covid-19 vaccine |  |  |
| Side effects |  |  |
| Not safe |  |  |
| Not effective |  |  |
| Covid-19 is not dangerous |  |  |
| Fear of infection |  |  |
| Against vaccination in general |  |  |
| Religious reason |  |  |
| Cultural reason |  |  |
| Belief in traditional remedies |  |  |

Appendix 2: Questionnaire (Bahasa Melayu version)

VAKSIN COVID-19: PENERIMAAN & KEBIMBANGAN

1. Kediaman sekarang (Sila tanda satu jawapan)

|  | Sarawak |
| --- | --- |
|  | Sabah |
|  | Perlis |
|  | Kedah |
|  | Pulau Pinang |
|  | Perak |
|  | Selangor |
|  | Melaka |
|  | Negeri Sembilan |
|  | Johor |
|  | Pahang |
|  | Kelantan |
|  | Trengganu |
|  | Wilayah Persekutuan Putrajaya |
|  | Wilayah Persekutuan Kuala Lumpur |
|  | Wilayah Persekutuan Labuan |
|  | Lain-lain: |

1. Tempat kelahiran (Sila tanda satu jawapan)

|  | Sarawak |
| --- | --- |
|  | Sabah |
|  | Perlis |
|  | Kedah |
|  | Pulau Pinang |
|  | Perak |
|  | Selangor |
|  | Melaka |
|  | Negeri Sembilan |
|  | Johor |
|  | Pahang |
|  | Kelantan |
|  | Trengganu |
|  | Wilayah Persekutuan Putrajaya |
|  | Wilayah Persekutuan Kuala Lumpur |
|  | Wilayah Persekutuan Labuan |
|  | Lain-lain: |

1. Umur (Tahun) (Sila tanda satu jawapan)

|  | 18-29 |
| --- | --- |
|  | 30-39 |
|  | 40-49 |
|  | 50-59 |
|  | 60 dan ke atas |

1. Jantina (Sila tanda satu jawapan)

|  | Lelaki |
| --- | --- |
|  | Perempuan |

1. Bangsa (Sila tanda satu jawapan)

|  | Melayu |
| --- | --- |
|  | Cina |
|  | India |
|  | Iban |
|  | Bidayuh |
|  | Melanau |
|  | Orang Ulu |
|  | Kadazan |
|  | Dusun |
|  | Murut |
|  | Bajau |
|  | Orang Asli |
|  | Lain-lain: |

1. Agama (Sila tanda satu jawapan)

|  | Islam |
| --- | --- |
|  | Kristian |
|  | Hindu |
|  | Buddha |
|  | Lain-lain: |

1. Status perkahwinan (Sila tanda satu jawapan)

|  | Berkahwin |
| --- | --- |
|  | Bercerai |
|  | Balu atau duda |
|  | Bujang |

1. Tahap pendidikan (Sila tanda satu jawapan)

|  | Sekolah rendah |
| --- | --- |
|  | Sekolah menengah |
|  | Pengajian tinggi (Kolej/Universiti) |
|  | Tiada pendidikan formal |

1. Pekerjaan (Sila tanda satu jawapan)

|  | Perubatan & Kesihatan |
| --- | --- |
|  | Pendidikan |
|  | Keselamatan & Pertahanan |
|  | Industri & Pembuatan |
|  | Pelancongan & Hospitaliti |
|  | Pembinaan |
|  | Pengurusan & Pentadbiran |
|  | Perkhidmatan |
|  | Pelajar |
|  | Pesara |
|  | Tidak bekerja |
|  | Lain-lain: |

1. Pendapatan bulanan (RM) (Sila tanda satu jawapan)

|  | 0-1200 |
| --- | --- |
|  | 1201-4000 |
|  | 4001-8000 |
|  | >8000 |

1. Penyakit (Sila jawap setiap kenyataan)

|  | Ya | Tidak |
| --- | --- | --- |
| Kencing manis |  |  |
| Darah tinggi |  |  |
| Kolesterol tinggi |  |  |
| Penyakit saluran pernafasan |  |  |
| Penyakit buah pinggang |  |  |
| Sakit jantung |  |  |
| Kanser |  |  |

1. Sumber maklumat tentang Covid-19 (Sila jawap setiap kenyataan)

|  | Ya | Tidak |
| --- | --- | --- |
| Media sosial (WhatsApp, Telegram, Twitter, Instagram, Facebook, Website etc) |  |  |
| Media massa (TV, Radio, Suratkhabar) |  |  |
| Petugas kesihatan |  |  |
| Rakan, keluarga, jiran dll |  |  |

1. Jika Kerajaan Malaysia memberi vaksin COVID-19 secara percuma, adakah anda bersetuju untuk menerima vaksinasi? (Sila tanda satu jawapan)

|  | Ya |
| --- | --- |
|  | Tidak, sila jawab soalan 14 |

1. Apakah kebimbangan anda tentang vaksin COVID-19? (Sila jawap setiap kenyataan)

|  | Ya | Tidak |
| --- | --- | --- |
| Kurang maklumat tentang vaksin Covid-19 |  |  |
| Kesan sampingan |  |  |
| Tidak selamat |  |  |
| Tidak berkesan |  |  |
| Covid-19 tidak berbahaya |  |  |
| Takut pada suntikan |  |  |
| Menolak vaksinasi secara umum |  |  |
| Faktor agama |  |  |
| Faktor budaya |  |  |
| Percaya dengan rawatan tradisional |  |  |
